# A case report of advanced pancreatic neuroendocrine carcinoma with Ki67 80%, CPS 0, and pMMR achieving durable complete response for over 7 years after combination immunotherapy

**DOI:** 10.3389/fimmu.2025.1682148

**Published:** 2025-12-01

**Authors:** Weiqiang Fan, Hui Xu, Yating Wu, Hang Li, Yonghai Peng

**Affiliations:** Department of Oncology, The CangShan District of the 900th Hospital, Fuzhou, Fujian, China

**Keywords:** immunotherapy, neuroendocrine carcinoma, probiotics, radiation therapy, case report

## Abstract

**Background:**

Pancreatic neuroendocrine carcinoma (pNEC) is a rare and aggressive malignancy. For patients with metastatic pNEC, the response to first-line chemotherapy remains unfavorable, with a median overall survival of less than one year. Furthermore, there are no standard-of-care therapeutic options following disease progression, representing a significant unmet clinical need.

**Case presentation:**

This is a case report of a patient diagnosed with metastatic pNEC presenting with upper abdominal pain, testing 80% positive for the Ki67 marker, a combined positive score (CPS) of 0, and proficient mismatch repair (pMMR). Following progression after chemotherapy, the patient received combination immunotherapy, involving multi-site, multi-dose, and multi-type radiotherapies combined with programmed death 1 (PD-1), accompanied by continuous probiotic supplementation. Imaging examination revealed that the tumor completely disappeared, and the patient maintained a no-evidence-of-disease (NED) status for over seven years.

**Conclusion:**

This case demonstrates that multi-site, multi-dose, and multi-type radiotherapies can more effectively reveal the heterogeneous tumor antigens, and the combined therapy of immunotherapy and probiotics may achieve sustained immune responses.

## Introduction

Pancreatic neuroendocrine carcinoma (pNEC) is relatively rare, accounting for 1-3% of pancreatic tumors. Currently, chemotherapy is the mainstay treatment for metastatic pNEC, but the median survival of patients receiving this therapy is less than one year, and there is no standard treatment following first-line therapy. In recent years, immunotherapy has shown great potential for cancer treatment, but pancreatic cancer responds poorly to it due to low immunogenicity and an immunosuppressive tumor microenvironment (TME) that characterizes these immunologically “cold” tumors (1). Efforts have been made to enhance the efficacy of immunotherapy in cold tumors by combining programmed death 1 (PD-1)/programmed death ligand 1 (PD-L1) with chemotherapy, anti-angiogenesis, and cytotoxic T lymphocyte antigen 4 (CTLA-4). However, the results are not satisfactory (2).

**Figure 1:**
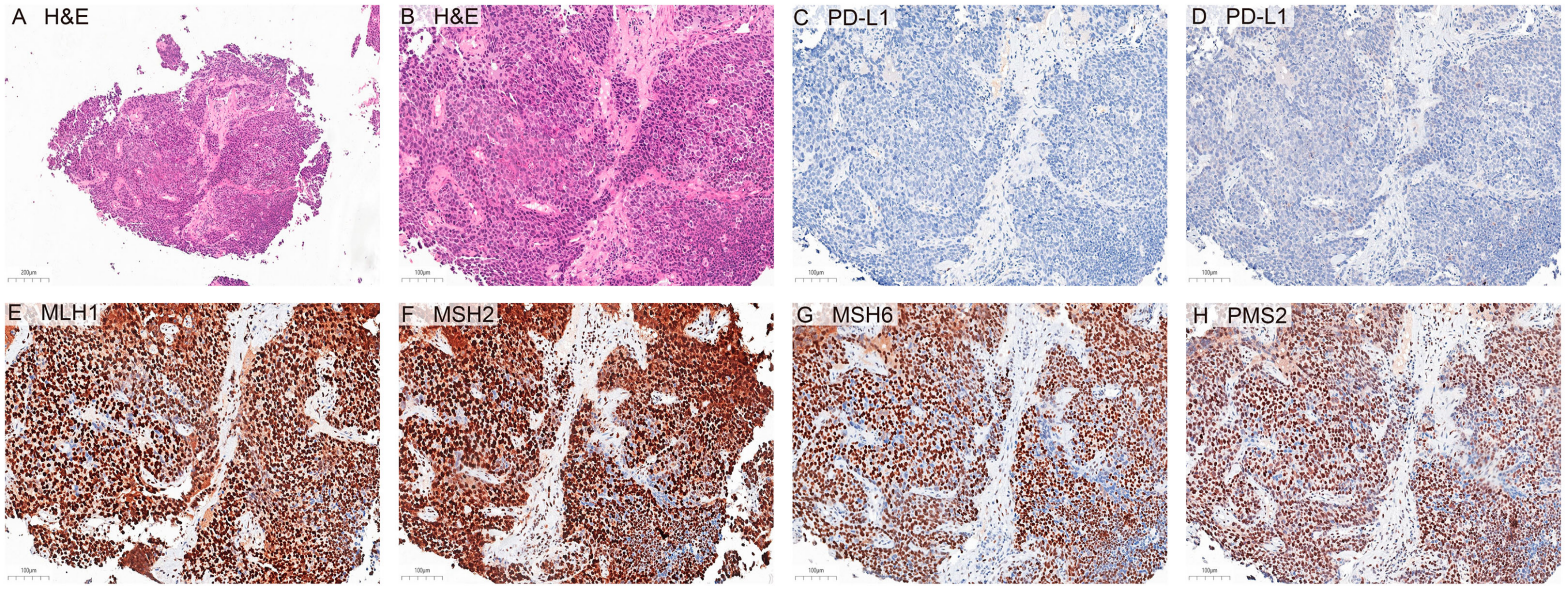
Histopathological and immunohistochemical examination of cervical lymph node metastasis. **(A)** Representative hematoxylin and eosin (H&E) staining images of metastatic tumor tissue. **(B)** Higher-magnification H&E demonstrating tumor morphology. **(C, D)** Immunohistochemical (IHC) analysis of PD-L1 expression, revealing negative staining in both immune cells **(C)** and tumor cells **(D)**, corresponding to a combined positive score (CPS) of 0. **(E-H)** Representative IHC images of DNA mismatch repair (MMR) proteins, demonstrating unaltered nuclear expression of MLH1 **(E)**, MSH2 **(F)**, MSH6 **(G)**, and PMS2 **(H)**, consistent with proficient MMR (pMMR) status. All images were recorded at 20× magnification (except A, 10×), with scale bars = 100 μm (shown in the lower-left corner of each panel).

Here, we report a case of a patient with metastatic pNEC [Ki67 80%, combined positive score (CPS) 0, proficient mismatch repair (pMMR)] (**Figure 1**, **Table 1**), which progressed following chemotherapy. Due to fear of prior chemotherapy, the patient requested a chemotherapy-free treatment regimen. Following immunological principles, we designed a treatment plan involving multi-site, multi-dose, and multi-type radiotherapies to induce heterogeneous tumor cells to release different tumor neoantigens. Building on this approach, PD-1 therapy was incorporated to restore T-cell function, and probiotic supplementation continued to enhance immunotherapy efficacy. Remarkably, the patient achieved complete radiographic tumor regression and has maintained a no-evidence-of-disease (NED) status over seven years.

**Table 1:** Immunohistochemical (IHC) markers.

Immunohistochemical marker	Staining localization	Result/Intensity
CD56	Membrane/Cytoplasm	+++
Synaptophysin (Syn)	–	Negative
Chromogranin A (CgA)	–	Negative
INSM1	–	Not performed
Ki-67	Nuclei	80%
TTF-1	Nuclei	+++
P53	Nuclei	90%
Villin	Cytoplasm	++
CKP	Cytoplasm	+++
CK7	–	Negative
CK20	–	Negative
PD-L1 (Dako 22C3)	–	CPS: 0
MLH1, MSH2, MSH6, PMS2	–	All Positive (pMMR)

The Ki-67 index was assessed according to the semi-quantitative standard (counting positive tumor cell nuclei), consistent with manual hot-spot evaluation methodology commonly used in pathological practice. INSMI was not evaluated in this case.

## Case presentation

**Figure 2:**
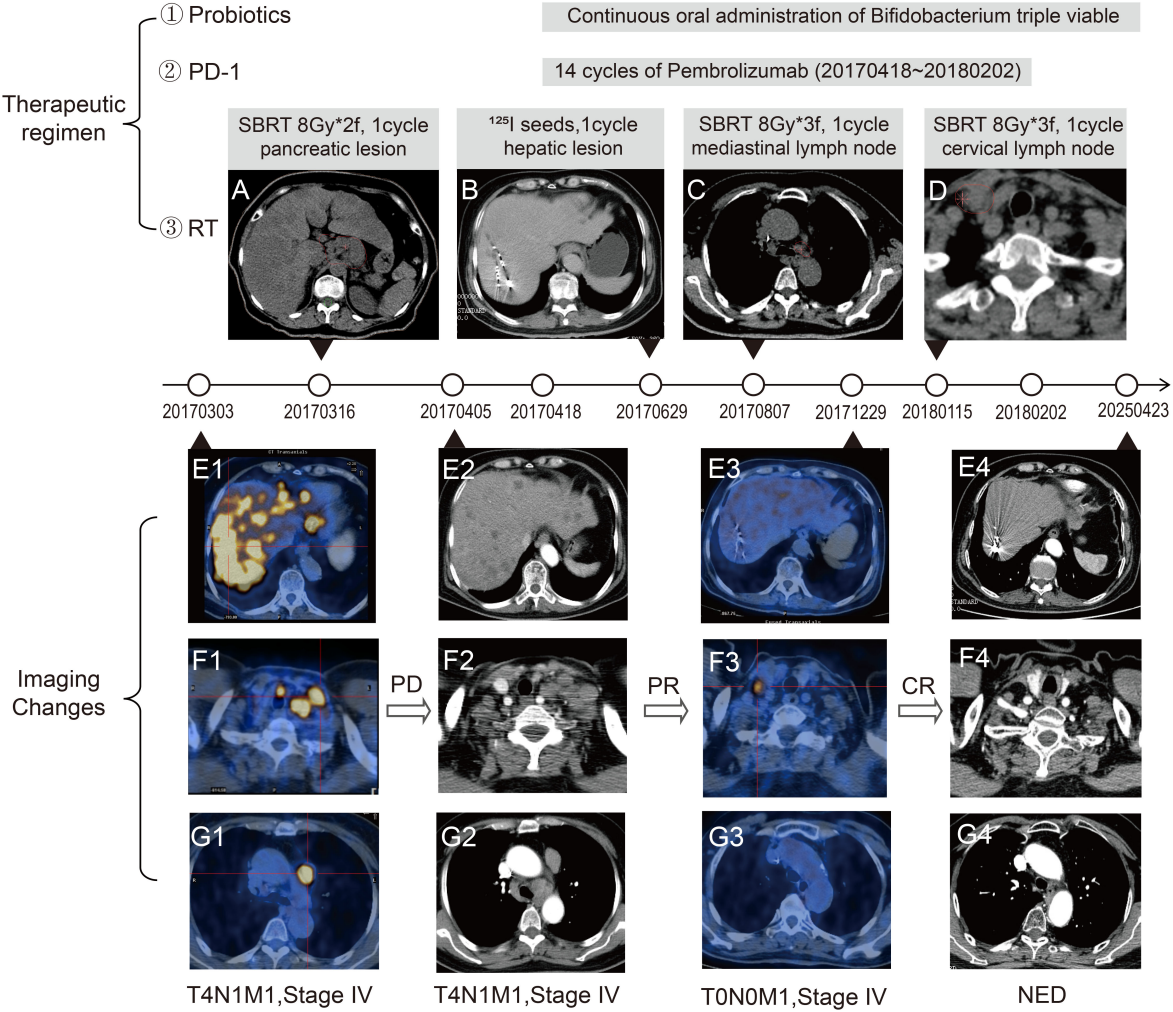
Treatment strategy and longitudinal imaging assessment of combined immunotherapy. **(A)** Delineation of radiation therapy target for the pancreatic lesion (red contour). **(B)** I-125 radioactive seed implantation site within the hepatic metastasis (white markers inside hepatic). **(C-D)** Radiotherapy planning for mediastinal **(C)** and cervical **(D)** lymph node metastases (red contour). **(E1-E4)** Serial imaging evolution of pancreatic and hepatic lesions during therapy. **(F1-F4)** Illustration of dynamic changes in cervical lymph node metastases at different treatment phases. **(G1-G4)** Temporal progression of mediastinal nodal disease. PET-CT fusion images **(E1, F1, G1, E3, F3, G3)** highlight metabolically active tumor foci (yellow areas).

A woman aged over 60 years visited our hospital in March 2017 with upper abdominal pain. Results of positron emission tomographic (PET)-computed tomographic (CT) imaging revealed pancreatic cancer with metastasis to the liver, mediastinum, and cervical lymph nodes (**Figures 2E1, F1, G1**; baseline and pre- and post-treatment images are shown in **Supplementary Figure S2**). Pathological examination of cervical lymph node biopsy confirmed Ki67 80%, CPS 0, and pMMR (MLH1+, MSH2+, MSH6+, PMS2+) (**Figure 1**; original report in **Supplementary Figure S3**). Based on these findings, a diagnosis of metastatic neuroendocrine carcinoma (NEC) was made, with a potential pancreatic origin, an Eastern Cooperative Oncology Group (ECOG) performance status of 1, and a body mass index (BMI) of 24.17 kg/m². Examination of the patient’s medical history revealed a 10-year history of hypertension and an 8-year history of diabetes, with no family history of genetic disorders. Physical examination showed no pronounced abnormalities. Considering the upper abdominal pain, the patient received stereotactic body radiation therapy (SBRT) for pancreatic lesions (dose: 16 Gy, 8 Gy × 2 fractions) (**Figure 2A**; the radiotherapy target area is shown in **Supplementary Figure S1A**). The number and size of lymph nodes in the neck and mediastinum increased following chemotherapy with etoposide and cisplatin (**Figures 2F2, G2**).

Subsequently, the patient received radioactive I-125 seed implantation for liver metastases (total of 36 seeds, activity: 0.6 mCi/seed) (**Figure 2B**), SBRT for mediastinal lymph nodes (dose: 24 Gy, 8 Gy × 3 fractions) (**Figure 2C**; the radiotherapy target area is shown in **Supplementary Figure S1B**), and SBRT for cervical lymph nodes (dose: 24 Gy, 8 Gy × 3 fractions) (**Figure 2D**; the radiotherapy target area is shown in **Supplementary Figure S1C**). She received 14 cycles of pembrolizumab therapy (200 mg intravenous infusion every three weeks) and long-term probiotic supplementation (Bifidobacterium triple viable capsule 6g bid) (**Table 2**). During treatment, the patient’s tumor continued to shrink. At the 13th cycle of PD-1 therapy, PET-CT scan showed complete response (CR) in the pancreatic, hepatic, and mediastinal lesions (**Figures 2E3, G3**). Notably, all lesions achieved CR following the 14 cycles of PD-1 therapy. No significant adverse events were observed during the treatment period. Blood glucose and blood pressure remained stable. The patient’s ECOG performance status was improved to 0, and BMI remained stable at 21.56 kg/m² after the treatment, and the patient showed good treatment compliance and tolerability. Further follow-up examinations demonstrated NED. In April 2025 (**Figures 2E4, F4, G4**), enhanced CT scans of the thoracic and abdominal regions exhibited NED, more than seven years following the termination of anticancer therapy.

**Table 2:** Timeline of diagnosis and treatment.

Timeline	Regimen/Occurrence	Detail	Duration	Cycles	Notes
2017-03	Definitive Diagnosis	pNEC (T4N1M1, Stage IV) Ki-67 80%, CPS: 0, pMMR	**-**	**-**	**-**
2017-03-08	Chemotherapy	Etoposide 100mg d1-3 + Cisplatin 30mg d1-3	3week	1	First-line PD
2017-03-16	SBRT	Site: pancreatic lesion; Dose: 16 Gy/2 fractions (8 Gy per fraction)	2day	1	Continuous Regression
2017-04-18	Probiotics	Bifidobacterium triple viable 6g bid	Continuous	Continuous	
2017-04-18	PD-1	Pembrolizumab 200 mg	1day	1/14	
2017-05-10	PD-1	Pembrolizumab 200 mg	1day	2/14	
2017-06-04	PD-1	Pembrolizumab 200 mg	1day	3/14	
2017-06-26	PD-1	Pembrolizumab 200 mg	1day	4/14	
2017-06-29	125I seeds	Site: hepatic lesio, 36 seeds, activity: 0.6 mCi/seed	1day	1	
2017-07-18	PD-1	Pembrolizumab 200 mg	1day	5/14	
2017-08-07	SBRT	Site: mediastinal lymph node; Dose: 24 Gy/3 fractions (8 Gy per fraction)	3day	1	
2017-08-11	PD-1	Pembrolizumab 200 mg	1day	6/14	
2017-09-05	PD-1	Pembrolizumab 200 mg	1day	7/14	
2017-09-27	PD-1	Pembrolizumab 200 mg	1day	8/14	
2017-10-19	PD-1	Pembrolizumab 200 mg	1day	9/14	
2017-11-10	PD-1	Pembrolizumab 200 mg	1day	10/14	
2017-12-14	PD-1	Pembrolizumab 200 mg	1day	11/14	
2017-12-29	PD-1	Pembrolizumab 200 mg	1day	12/14	
2018-01-15	SBRT	Site: mediastinal lymph node; Dose: 24 Gy/3 fractions (8 Gy per fraction)	3day	1	
2018-01-18	PD-1	Pembrolizumab 200 mg	1day	13/14	
2018-02-02	PD-1	Pembrolizumab 200 mg	1day	14/14	CR

pNEC, pancreatic neuroendocrine carcinoma; SBRT, stereotactic body radiotherapy; PD, progressive disease; CR, complete response.

## Discussion

In this case of advanced pNEC with a Ki67 index of 80% after chemotherapy, despite CPS 0 and pMMR (cold tumor), the patient achieved CR after 14 cycles of pembrolizumab + multi-site heterogeneous radiotherapies + long-term oral probiotics. Following the initial CR, the patient did not undergo any further anticancer therapy. Remarkably, routine follow-ups over the next seven years consistently showed NED.

The synergistic potential of radiotherapy and immunotherapy has been reported in several NECs, including cervical, esophageal, and pulmonary (3–5). The strategy of integrating localized radiotherapy with immunotherapy in these NEC cases is highly compelling. However, there is limited clinical evidence on the widespread application of this combination regimen in NECs. While some meta-analyses have demonstrated survival benefits from adding thoracic radiotherapy to standard chemoimmunotherapy in extensive-stage small cell lung cancer (6, 7), gaps remain in prospective randomized controlled trials directly evaluating the combination of radiotherapy and immune checkpoint inhibitors (ICIs) in the broader NEC population. More critically, key clinical questions in this field remain unaddressed: there is currently no consensus on the optimal treatment sequence (concurrent versus sequential) or the most immunogenic radiotherapy dose and fractionation schedule (hypofractionated versus conventional) (8, 9). The present case provides several novel perspectives on this paradigm. First, unlike most reported cases that employed single-site or standardized radiotherapy, the patient in our case report received multi-site, multi-dose, and multi-type radiotherapies, which may more comprehensively reveal the heterogeneous antigenic landscape of the tumor and potentiate a broader T-cell response. Second, despite CPS of 0 and pMMR status—biomarkers typically associated with limited immunotherapy response—the patient achieved a complete and durable remission, suggesting that such radiological diversity may help overcome immunoresistant profiles. Third, the prolonged administration of probiotics alongside combination therapy represents an underexplored adjuvant strategy that may modulate the gut-immune axis and sustain antitumor immunity (10). Finally, achieving the NED status for over seven years in metastatic pNEC is extremely. It highlights the potential of this integrated approach to facilitate long-term immune control beyond what has been previously described with conventional radio-immunotherapy combinations.

Clinically, it is challenging to transfer cold tumors into hot tumors, and researchers are testing the potential of immunotherapy to improve cancer outcomes. The combination of radiotherapy and immunotherapy is one direction that needs to consider radiotherapy dose, number of irradiation sites, tumor biology, and immune status, necessitating the development of individualized treatment plans. Currently, radiotherapy is considered a group of diverse drugs with varying efficacy and toxicities (11, 12). Our treatment approach began with multi-site SBRT and I-125 seed implantation to release antigens from heterogeneous tumor locations, followed by PD-1 therapy to restore T-cell function and sustained oral probiotics to boost immunotherapy efficacy. This sequential approach leverages the potential of radiotherapy to induce immunogenic cell death and reshape the TME, thereby priming a systemic anti-tumor immune response that may synergize with subsequent immune checkpoint inhibition. Complete exposure of heterogeneous tumor antigens is a key step in initiating immune responses. Hypofractionated radiotherapy can cause DNA double-strand breaks, thus inducing tumor cell apoptosis and the release of tumor-specific antigens. When combined with immunotherapy, hypofractionated radiotherapy can also exert distant effects. Evidence has shown that the 8Gy*3f fractionated regimen can yield substantial synergistic immunotherapeutic effects (13). The distant effect of SBRT + immunotherapy has been observed in animal models and clinical studies (14). In view of the heterogeneity of the immune microenvironment and the difference in antigen expression at diverse tumor sites, studies have shown that multi-site radiotherapies can more effectively enhance the efficacy of immunotherapy than single-site radiotherapies (15). In the study by Justin involving a mouse tumor model, the results indicated that the dose-differentiated radiotherapy promoted the clonal expansion of effector CD8 T cells, and when combined with ICIs, heterogeneous radiotherapy yielded stronger antitumor immune responses than homogeneous radiotherapy (16). I-125 radioactive seed, which emits both X-rays and γ-rays with a half-life of 59.4 days, provides sustained tumor antigen exposure. The gut microbiota plays a critical role in modulating both innate and adaptive immunity, as well as reshaping the TME—factors that collectively influence immunotherapy efficacy (17). In a clinical study comprising nivolumab + ipilimumab for advanced renal cell carcinoma, the experimental group was only administered with additional probiotics, which caused a significant fourfold increase in progression-free survival (12.7 months versus 2.5 months, hazard ratio 0.15, 95% confidence interval 0.05-0.47, P = 0.001) (18). Based on these findings, we designed the present protocol. The immunological mechanisms underlying this exceptional response—specifically, that radiotherapy may have released heterogeneous neoantigens and that probiotics may have modulated gut immunity—must be considered speculative. Although these hypotheses are plausible and supported in other malignancies, they require rigorous validation in prospective studies.

The abscopal effect, a phenomenon of systemic tumor regression induced by localized radiotherapy, represents a promising avenue for enhancing antitumor immunity. However, this effect in gastrointestinal neuroendocrine tumors (GI-NETs) has not been well studied. It may be attributed to the generally low immunogenicity of GI-NETs and their complex disease spectrum (19, 20). Encouragingly, the underlying mechanisms of the abscopal effect—including radiotherapy-induced antigen release and T-cell priming—are broadly applicable (21). Moreover, clinical synergistic effects between radiotherapy and ICIs observed in adjacent gastrointestinal cancers (22) provide a rational basis for investigating similar strategies in GI-NETs. Notably, combination immunotherapy (e.g., ipilimumab/nivolumab) has shown substantial activity in high-grade GI-NETs, underscoring the potential for immune activation in a subset of patients (23).

This probiotic formulation consists of lyophilized live bacteria of three species: *Bifidobacterium longum*, *Lactobacillus bulgaricus*, and *Streptococcus thermophilus*. As this is an exploratory study, the rationale for selecting this regimen is based on several considerations: its putative immunomodulatory synergy, established clinical accessibility, and palatability—a key factor for ensuring long-term patient adherence. Growing evidence highlights that the gut microbiome is a key regulator of response to ICIs and enhances innate immunity (24–26). The core mechanistic driver may be *Bifidobacterium longum*, which has been demonstrated to directly enhance anti-tumor CD8+ T cell responses by activating dendritic cells for superior antigen presentation and T cell priming (27). Furthermore, clinical data link the abundance of *B. longum* with favorable outcomes in melanoma patients receiving anti-PD-1 therapy (28). *Lactobacillus bulgaricus* generates an exopolysaccharide (EPS-R1) that specifically increases CCR6+ CD8+ T cells. Following expansion in Peyer’s patches, these cells migrate to the TME that expresses the cognate chemokine CCL20, where they directly enhance anti-tumor activity. This mechanism significantly augments the efficacy of ICIs (29, 30). Despite limited direct evidence for *Streptococcus thermophilus* in ICI contexts, its presence has been correlated with positive clinical responses in ICI-treated patients (31), suggesting a potential role in maintaining a favorable gut ecological niche. Collectively, this probiotic formulation is postulated to work not as a simple aggregate but as a synergistic consortium: *B. longum* serves as the primary engine for direct T-cell activation, while *L. bulgaricus* and *S. thermophilus* contribute to shaping a robust, systemically Th1-polarized immune background. This multi-faceted immunomodulation, targeting both the adaptive effector arm and the innate immune landscape, provides a convincing mechanistic basis for the observed enhanced efficacy of ICIs and warrants further clinical investigation. The lack of standardization for the Bifidobacterium triple viable protocol is a recognized limitation of this exploratory research, highlighting the need for method optimization in future studies. Notably, despite multiple studies on the synergistic effects of probiotics with immunotherapy, current studies on Bifidobacterium triple viable primarily focus on its role in improving gastrointestinal functions in gastroenteritis, diarrhea, and radiation-induced enteritis.

Following the successful diagnosis of advanced pNEC with Ki67 80%, the patient consulted multiple oncologists, all of whom predicted a dismal survival rate. Interestingly, the patient maintained the NED status (currently exceeding 7 years) and achieved complete daily living ability after treatment. The patient, who described her recovery as a miracle, generously consented to share her medical records. This case prompted us to conduct an exploratory retrospective study of EGFR/ALK wild-type, PD-L1-negative metastatic NSCLC, and a prospective study of advanced HCC. In these studies, promising outcomes were achieved with a combination therapy comprising SBRT(8Gy*3f), PD-1 inhibitors, and probiotics (32, 33). Nonetheless, the underlying mechanisms remain unexplored, which presents a major limitation of our current strategy and a critical direction for our future research. Additionally, despite the promising efficacy of multimodal therapy incorporating ICIs observed in this case and our studies, their broad clinical application necessitates further validation through large-scale, prospective randomized controlled trials and a deeper exploration of the underlying mechanisms. In analyzing this case, we observed memory immune responses post-treatment; however, a key limitation of this case report is the absence of additional pathological specimens, which were not documented at the time (eight years prior), thereby precluding deeper exploration into the underlying mechanisms of the observed therapeutic effect. Notably, the proposed mechanism involving memory immune responses remains hypothetical due to the lack of immunologic biomarker data (e.g., lymphocyte subsets, cytokines, circulating tumor DNA) during the follow-up period. This absence restricts our ability to fully elucidate the immunological mechanisms underlying the observed remarkable response.

## Conclusion

In the present case, we demonstrate that combining immunotherapy with multi-site, multi-dose, and multi-type radiotherapies, along with long-term probiotic supplementation, may transform cold tumors into hot tumors in patients with advanced pNEC. This strategy may activate the immune system, resulting not only in complete remission but also memory immune responses, which together contribute to sustained therapeutic benefits.

## Data Availability

The original contributions presented in the study are included in the article/**Supplementary Material**. Further inquiries can be directed to the corresponding author.

## Supplementary material

Supplementary Figure 1 Delineation of gross tumor volume in pancreatic and lymph node metastases.

Supplementary Figure 2 Alterations in serial images in cervical lymphadenopathy, mediastinal lymphadenopathy, pancreatic lesions, and hepatic metastases.

Supplementary Figure 3 Immunohistochemical characterization of cervical lymph node biopsy specimens.
