# Supplementary material for: A case report of advanced pancreatic neuroendocrine carcinoma with Ki67 80%, CPS 0, and pMMR achieving durable complete response for over 7 years after combination immunotherapy
Source: Front Immunol. 2025 Dec 1;16:1682148. doi: 10.3389/fimmu.2025.1682148 (PMC12702980; doi:10.3389/fimmu.2025.1682148)
Supplement: Supplementary Figure 1 — Delineation of gross tumor volume in pancreatic and lymph node metastases. [file DataSheet3.pdf]

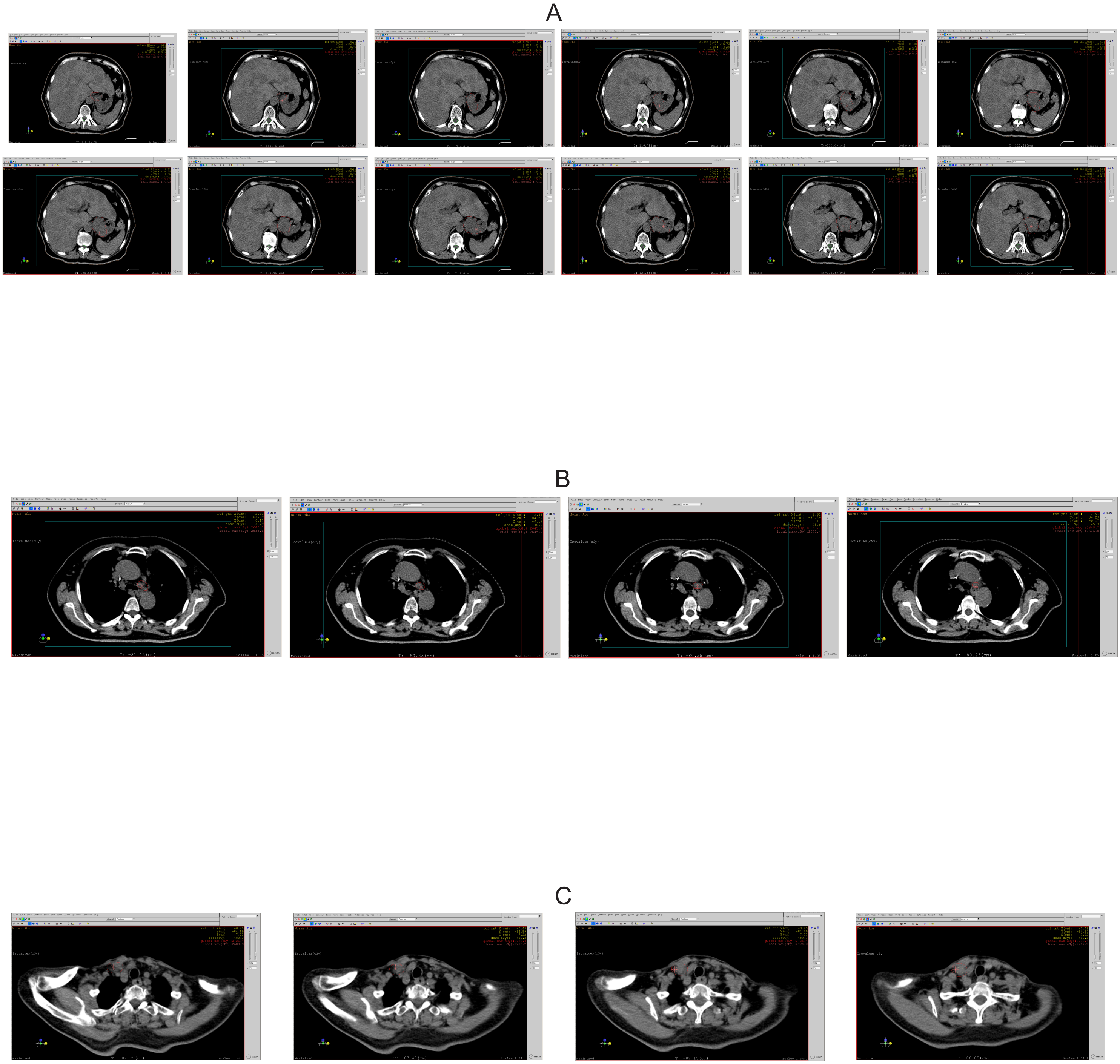

Figure S1. Gross tumor volume (GTV) delineation in pancreatic and lymph node metastases. (A) Pancreatic primary tumor GTV (red contour). (B) Mediastinal lymph node metastasis GTV (red contour). (C) Cervical lymph node metastasis GTV (red contour).
