# Supplementary material for: A case report of advanced pancreatic neuroendocrine carcinoma with Ki67 80%, CPS 0, and pMMR achieving durable complete response for over 7 years after combination immunotherapy
Source: Front Immunol. 2025 Dec 1;16:1682148. doi: 10.3389/fimmu.2025.1682148 (PMC12702980; doi:10.3389/fimmu.2025.1682148)
Supplement: Supplementary Figure 2 — Alterations in serial images in cervical lymphadenopathy, mediastinal lymphadenopathy, pancreatic lesions, and hepatic metastases. [file DataSheet2.pdf]

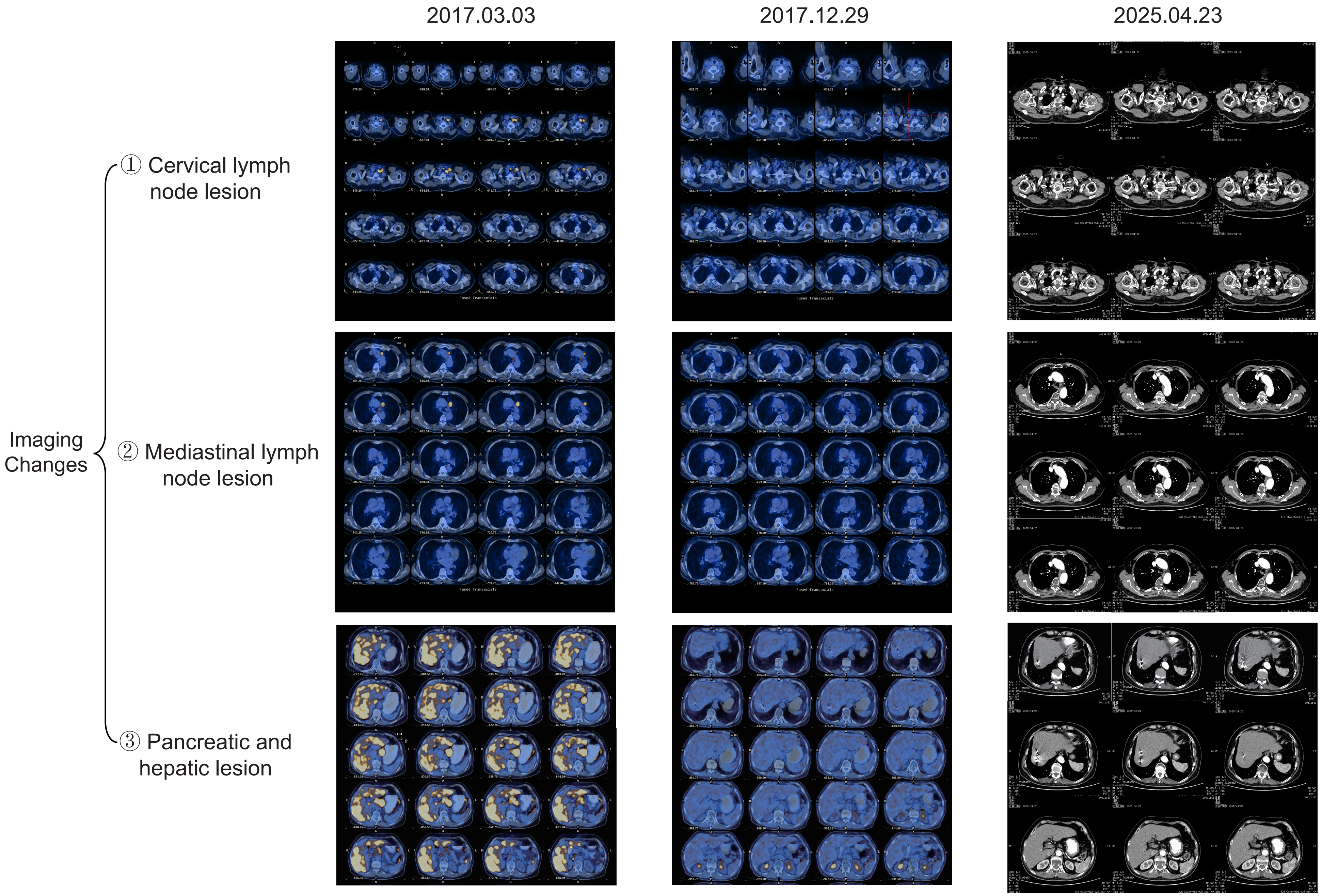


Figure S2 Serial imaging changes in cervical lymphadenopathy, mediastinal lymphadenopathy, pancreatic lesions and hepatic metastases
