# Supplementary material for: A case report of advanced pancreatic neuroendocrine carcinoma with Ki67 80%, CPS 0, and pMMR achieving durable complete response for over 7 years after combination immunotherapy
Source: Front Immunol. 2025 Dec 1;16:1682148. doi: 10.3389/fimmu.2025.1682148 (PMC12702980; doi:10.3389/fimmu.2025.1682148)
Supplement: Supplementary Figure 3 — Immunohistochemical characterization of cervical lymph node biopsy specimens. [file DataSheet1.pdf]

190679

[redacted]

免疫组化病理诊断报告书

[redacted]

院别：

病理号：[redacted]

姓名 [redacted] 性别 女 年龄 65Y 联系电话 [redacted] 收到日期 2017-03-09

科别 肿瘤科 床号 [redacted] ID号 [redacted] 送检医师 [redacted] 制片技师 [redacted]

临床诊断 待查 检测样本 免疫组织化学染色诊断-仪器法.

恶性肿瘤靶向治疗及预后标记物染色结果（意义见背页）

抗体类型	着色部位	范围	说明
Villin	浆	++	
CD56	膜/浆	+++	
CKP	浆	+++	
P53	核	90%	
TTF-1	核	+++	
Ki-67	核	80%	

阴性抗体类型：CK7、CK20、CgA、Syn

免疫组化半定量标准：无着色或零星散在(<1%)阳性为阴性，<25%肿瘤细胞阳性为+，阳性浸润炎症细胞/淋巴细胞（瘤）覆盖病灶的面积<25%为+，25~49%++，50~75%+++，>75%++++；ER、PR、P53、Ki-67计肿瘤细胞核阳性率。

【PD-L1免疫组化检测】

1.检测信息：抗体克隆号：Dako 22C3；检测平台：Dakolink48；质控样本：合格；肿瘤细胞数量充足：≧100个；评分标准：Dako 22C3评分标准.

2.检测结果：肿瘤及免疫细胞PD-L1表达水平CPS：0。

3.PD-1（NAT）：淋巴细胞中阳性数量0个/HPF（热点区）。

【dMMR筛查】MLH1阳性，MSH2阳性，MSH6阳性，PMS2阳性（注：＜5%为阴性）；提示本例肿瘤不存在错配修复功能缺陷（pMMR）。

病理诊断：

[redacted]

“左颈部肿物穿刺标本”：免疫组化支持转移性神经内分泌癌，请结合临床查找原发灶。

[redacted]

Figure S3 Immunohistochemical Characterization of Cervical Lymph Node Biopsy Specimens
